# Proteomic analysis of serum from a MeCP2 patient reveals an arginine biosynthesis pathway affected by the p.Lys254* variant

**DOI:** 10.1002/ccr3.9503

**Published:** 2024-11-29

**Authors:** Xiaoqin Gong, Tuanmei Wang, Anji Chen, Geng Ouyang, Mengmei Lv, Jianxin Gao, Baomei Yu, Min Wu, Huaxue Qi, Yunsu Zhu, Jinjin Dai, Jun He, Jiyang Liu, Xiangwen Peng

**Affiliations:** ^1^ Hunan Provincial Key Laboratory of Regional Hereditary Birth Defects Prevention and Control Changsha Hospital for Maternal and Child Health Care Affiliated to Hunan Normal University Changsha China; ^2^ Changsha Municipal Health Commission Changsha China

**Keywords:** heart defect, Kett syndrome, MeCP2, metabolism, whole‐exome sequencing

## Abstract

**Key Clinical Message:**

This study reports a Chinese male patient with a novel MeCP2 p.Lys254*variant. Upon birth, the patient presented with typical symptoms, such as abnormal electroencephalogram, immature sleep rhythm, hypotonia, feeding difficulties, pulmonary fluid accumulation, horizontal fissures in the lungs, hypoventilation, and heart defects.

**Abstract:**

MeCP2 is a gene located on the X chromosome and the main pathogenic gene responsible for Rett syndrome, which mainly occurs in females. Herein, we identified a male patient with a novel MeCP2 p.Lys254* variant through whole‐exome sequencing, although both parents are wild type. Upon birth, the patient presented with typical symptoms, such as abnormal electroencephalogram, immature sleep rhythm, hypotonia, feeding difficulties, pulmonary fluid accumulation, horizontal fissures in the lungs, hypoventilation, and other symptoms. Period of breathing support, but also found that the boy had a heart defect and horizontal fissure in the lungs. Our discovery of a new spontaneous MeCP2 nonsense mutation enriches the understanding of Rett syndrome and provides a reference for its early diagnosis and treatment.

## 1 INTRODUCTION

Methyl CpG binding protein 2 (MECP2) (OMIM *300005) is located at Xq28 and is a multifunctional gene with ubiquitous expression. MeCP2 possesses many functions, such as orchestrate chromatin compartmentalization and higher order genome architecture.^1^ MeCP2 is necessary for normal neuronal function and overall health throughout the lifespan.^1^ Mutations in MECP2 can lead to a wide spectrum of clinical presentations that range from mild intellectual impairment to severe neonatal encephalopathy and premature death.^2^ Female MeCP2–deficient mice become hypoactive and exhibit motor and breathing defects after 3 months of age.^3^ MeCP2 is a DNA‐binding protein involved in higher order chromatin organization and RNA splicing. Mutations in MeCP2 virtually affect all the organs and tissues,^2^ including metabolic anomalies, such as reduced N‐acetylaspartate, myo‐inositol, glutamine plus glutamate,^4^ cholesterol metabolism,^5^ liver lipid metabolism,^6^ tyrosine metabolism,^7^ platelet activation, and respiratory metabolism.^8^ However, the molecular functions of MeCP2 in humans remain unclear. To find additional molecular functions of MeCP2 in humans, the serum proteome of a patient with the MeCP2 p.Lys254* variant was analyzed using tandem mass tag (TMT)‐labeled quantitative proteomics. Similar to observations in null mutation mice, our analysis revealed considerable changes in platelet activation, tyrosine metabolism, fatty acid degradation, and metabolism‐related proteins.^7^, ^9^ Moreover, we observed substantial changes in carbon metabolism, arginine biosynthesis, and glycolysis. Our discovery of a new spontaneous MeCP2 nonsense mutation enriches the knowledge of Rett syndrome and provides a reference for its early diagnosis and treatment.

## 2 CASE HISTORY

**FIGURE 1:**
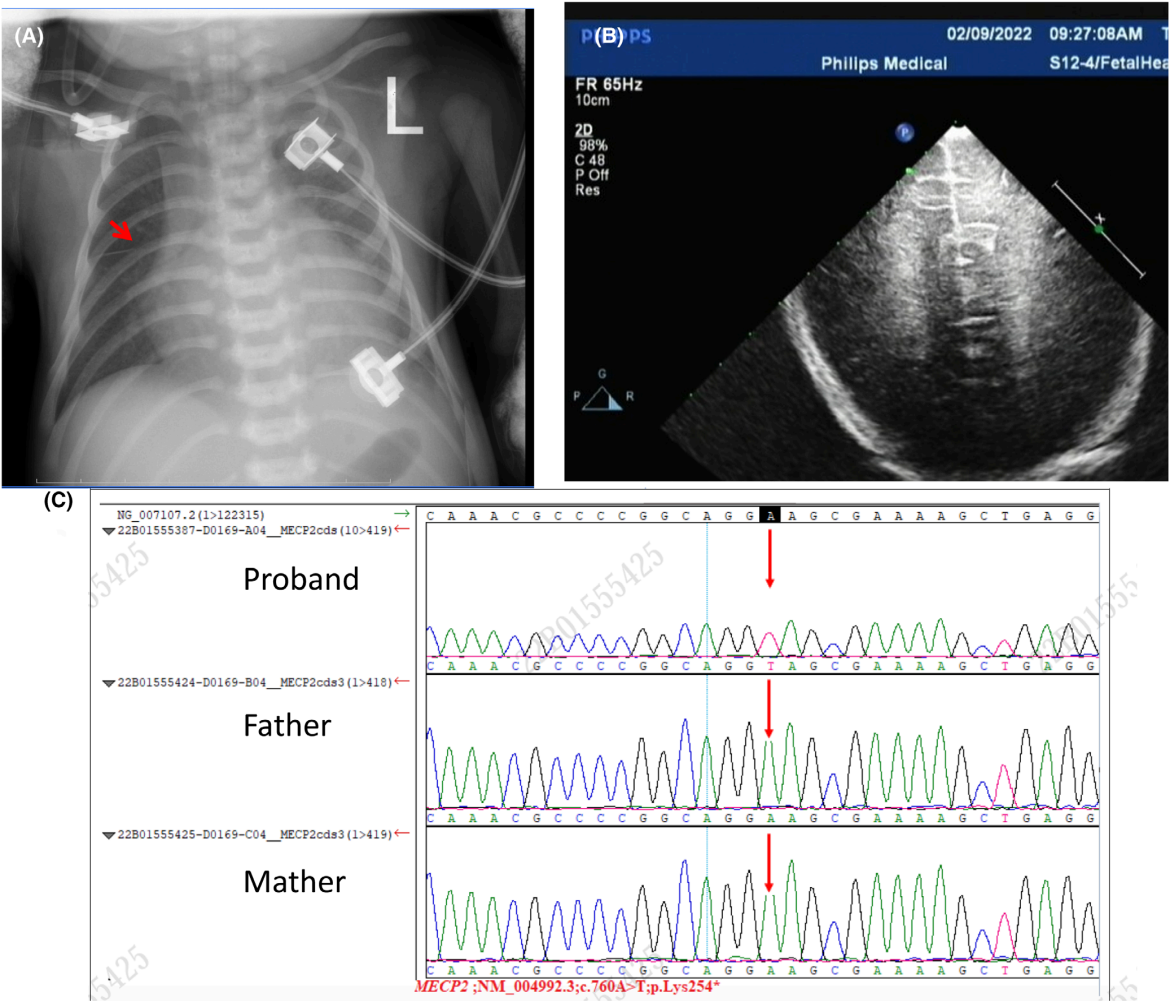
Phenotype of the proband. (A) D2 Chest x‐ray shows that the transparency of both lungs is slightly low; the markings of both lower lungs are blurred with horizontal fissures (red arrow). (B) Echocardiography shows that the foramen ovale is shunted from left to right (2.8 mm) with mild mitral and tricuspid regurgitation in the proband. (C) First‐generation sequencing verifies disease‐causing genes in the proband and family members: The MeCP2 p.Lys254* variant.

**FIGURE 2:**
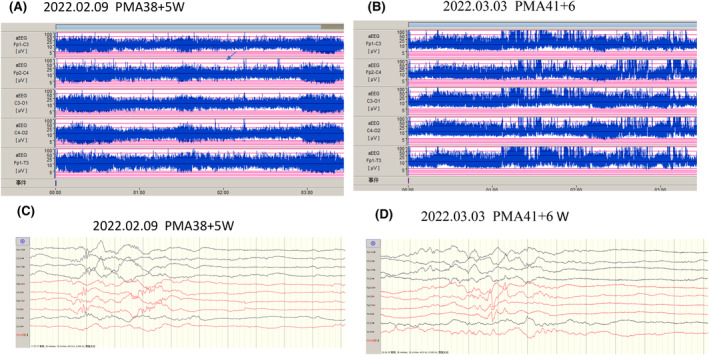
Brain function–monitoring amplitude‐integrated EEG (aEEG) indicates that the proband's sleep cycle is not matured. (A, C) Brain function–monitoring aEEG at 2022.02.09 (PMA38 + 5 W). (B, D) Brain function–monitoring aEEG at 2022.03.03 (PMA41 + 6 W).

With a gestational age of 38 weeks and 4 days, the mother of the patient delivered in the obstetrics department of our hospital on February 8, 2022. The infant was born at 2.55 kg following a 5‐h rupture of membranes with amniotic fluid++; the umbilical cord was wrapped around the neck for 1 week prior to delivery. Ashley score: 1 min‐8 points; Poor reaction, weak sucking, poor muscular tension, small for gestational age, weak crying, high PCO_2_, ventilator‐assisted ventilation, positive pressure ventilation of resuscitation capsule at birth for 1 min, 5–10, hypothermia; Dysphagia, increased and blurred bilateral lung markings (Figure 1A), at 4 days color Doppler echocardiography revealed that the foramen ovale shunted from left to right (2.8 mm) with mild mitral and tricuspid regurgitation (Figure 1B); the parents were normal with no family genetic history. Then, the whole exome of peripheral blood was sequenced, revealing that the proband possessed a mutation in the MeCP2 gene (NM_004992). P3: c.760A > T (p.Lys254 *) variation, but parents are normal (Figure 1C). Brain‐function monitoring shows sleep–wake cycle: yes, but immature sleep (Figure 2A,B). The interval between two electroencephalogram (EEG) sessions was 3 weeks, revealing continuous maturation of the EEG patterns; however, no considerable improvement was observed in the patient (Figure 2C,D).

### 2.1 Treatment

#### 2.1.1 Main treatment process

After birth, the child exhibited poor mental reactions, no crying, frowning, minimal movement, shallow breathing, and an inability to tolerate deoxygenation. In terms of respiration, he was given oxygen through a nasal catheter for 4 days. On the 12th day, he was transferred to our department from the second new department and switched to noninvasive ventilator–assisted ventilation (NIPPV) for 10 days considering the following parameters: PIP: 20 cmH_2_O, PEEP: 6 cmH_2_O, RR: 40 times/min, FiO_2_: 25%, and Ti: 0.6 s. After the ventilator was stopped on the 22nd day, the blood oxygen saturation of the child decreased (from 98% to 88%). On the 24th day, the noninvasive partial pressure of carbon dioxide was measured and the partial pressure of carbon dioxide increased to 67 mmHG, and the child was given NIPPV again (FiO_2_: 21%, PIP: 15 cmH_2_O, PEEP: 4 cmH_2_O, RR: 40 times/min, and Ti: 0.55 s). On March 1, caffeine citrate was added to stimulate the child's breathing, and the child demonstrated intolerance to weaning from support and transitioning to high‐flow oxygen. After these adjustments, carbon dioxide was retained. At present, considering the current parameters, blood oxygen levels can be maintained, breathing is stable, and oral secretions are abundant.

#### 2.1.2 Infection

After admission, the neutral ratio was on the higher side for three consecutive days, and the results of cerebrospinal fluid were normal (2.10, 2.23). After 7 days of anti‐infection treatment with cefotaxime, the infection indicators were normal.

#### 2.1.3 Feeding

After birth, the neonate underwent a 3‐day fasting period to rule out genetic metabolic diseases. On the fourth day after birth, the milk supply reached full capacity, and the early sucking force was weak; thus, training on swallowing and sucking was provided, and self‐sucking was completed.

#### 2.1.4 Nervous system

In the early stage, the infant exhibited minimal crying and spontaneous movement. At present, crying is present only in response to painful stimuli (elicited twice), with incomplete grasping and hugging reflexes and no observed episodes of screaming or convulsions; however, sucking and foraging can be induced.

#### 2.1.5 Physical examination

Magnetic resonance imaging of the head did not reveal any abnormalities; however, the EEG exhibited mild to moderate abnormalities. With regard to genetic metabolism, no abnormality was observed during the initial screening and reexamination of neonatal disease, and no abnormality was found in No. 13 urine organic acid; Thyroid function: TSH 0.84 mIU/l, FT4: 13.85 pmol/L, started taking levothyroxine tablets 5 μg/kg. d on the 13th, and thyroid function was normal by color Doppler ultrasound. The thyroid function recheck half a month showed that thyrotropin: 0.16 mIU/l, free triiodothyronine: 5.21 pmol/L, free thyroxine: 19.40 pmol/L, indicating that TSH was low, FT4 was normal.

#### 2.1.6 Discharge condition

Under NIPPV (FiO_2_: 21%, PIP: 15 cmH_2_O, PEEP: 4 cmH_2_O, RR: 40 times/min, and Ti: 0.55 s), the patient maintained percutaneous oxygen saturation and stable respiratory effort. No fever, convulsions, vomiting, and abdominal distension were observed. The patient drank 60 mL of q3h milk every day; stool and urine output were normal. Physical examination: T: 36.8°C, R: 43 times/min, HR: 141 times/min, sPO_2_; 94%, wt: 2.99 kg, poor mental reaction, independent activity, flat and soft anterior fontanel, less ruddy skin, less ruddy mouth and lip, no congestion in the pharynx, soft neck, clear respiratory sound of both lungs, no rale heard. The examination revealed a normal heart rhythm (uniform and strong without murmurs); the abdomen was flat and soft; the liver and spleen did not expand; the navel was dry and clean; the umbilical wheel was not red and swollen; the bowel sounds were normal; the limbs were warm; the muscle tension of both upper limbs was normal; the muscle tension of both lower limbs was low. The cuddle reflex was incomplete; however, the holding reflex, sucking reflex, and foraging reflex could be induced.

### 2.2 TMT‐labeled quantitative proteomics shows many proteins changed in patients

**FIGURE 3:**
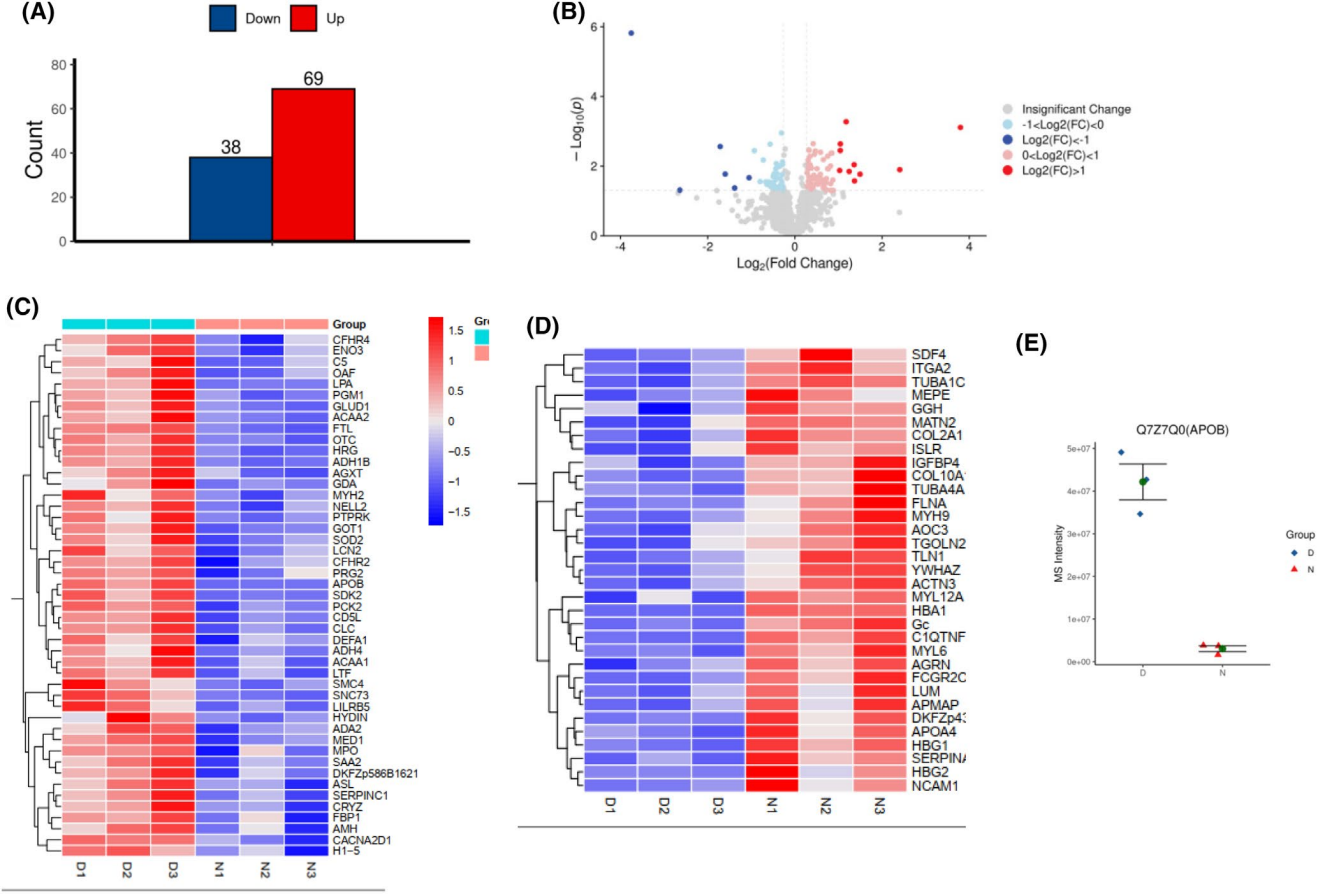
Detection of the serum of the proband and normal infant through TMT protein mass spectrometry (removal of high‐expression protein). (A) Number of up‐expression and down‐expression proteins. (B) Volcanic map of differential proteins. (C, D) Clustering heat map of differential proteins in two groups of samples. The color depth indicates the relative expression level. (E) Scatter plot of the differential protein APOB expression.

**FIGURE 4:**
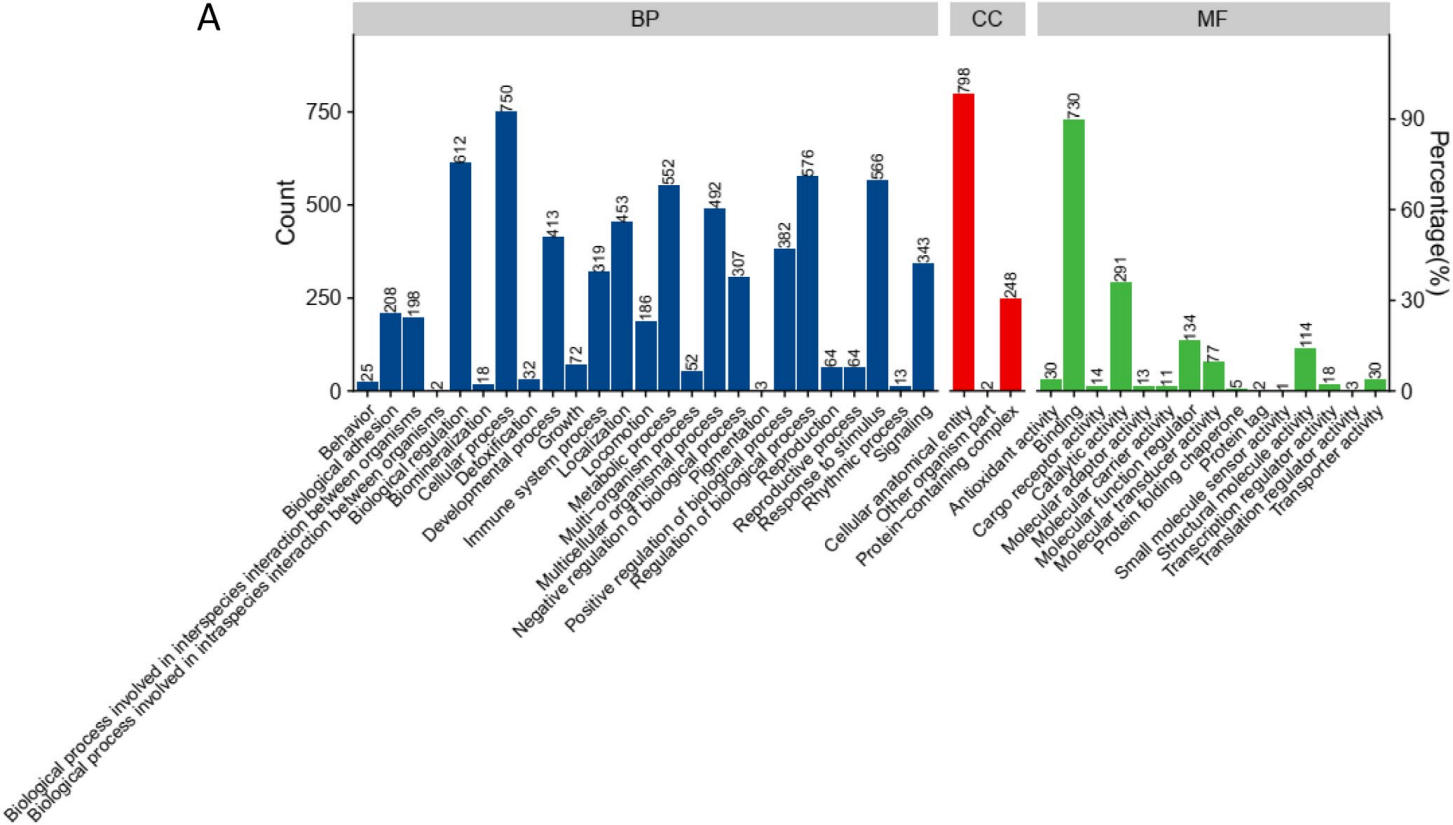
(A) GO term annotations of differential proteins.

To determine which protein considerably influences the patient, the serum proteome that removed highly expressed proteins of the patients was analyzed using TMT‐labeled quantitative proteomics. The analysis revealed differential protein expression in the patient compared to controls (three children of the same age). We identified 38 downregulated and 69 upregulated proteins (Figure 3A–E). The APOB protein exhibited considerable changes. We used the gene ontology (GO) database to describe the differentially expressed proteins on the basis of their biological process (BP), molecular function (MF), and cellular component (CC). With regard to the BP, the differential proteins are mainly associated with biological regulation (53 proteins), cellular process (70 proteins), metabolic process (50 proteins), and reproductive process (54 proteins); in terms of the MF, the proteins are mainly associated with binding (69 proteins) and catalytic activity (35 proteins). In terms of the CC, the proteins are mainly associated with cellular anatomical entities (75 proteins; Figure 4A).

**FIGURE 5:**
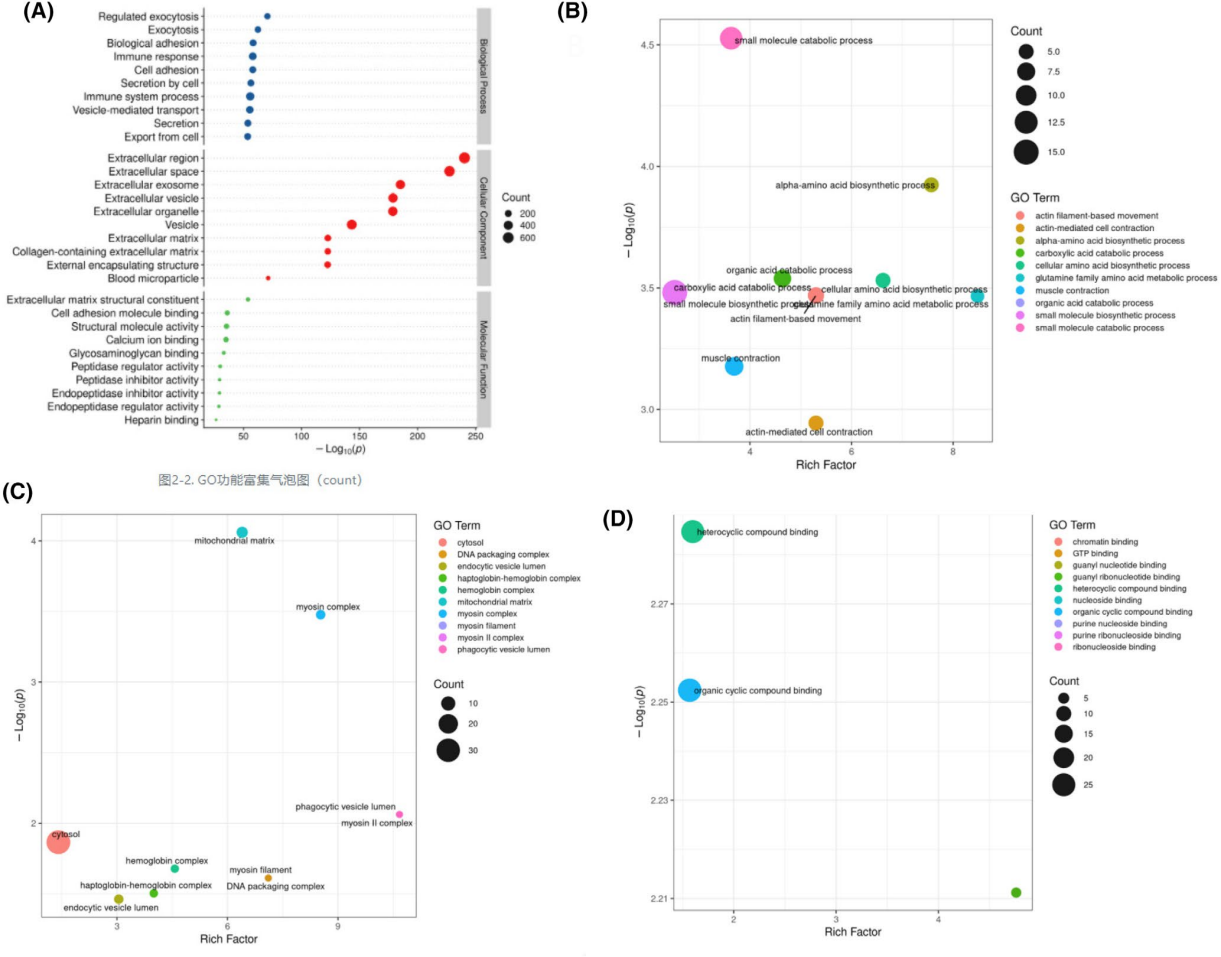
GO function annotation and enrichment of differential proteins. (A) GO function enrichment analysis of all differential proteins. (B) GO function enrichment analysis of the BF term (top 10). (C) GO function enrichment analysis of the CC term (top 10). (D) GO function enrichment analysis of the MF term (top 10).

To further screen the GO terms that are statistically important, we conducted Fisher's exact test for enrichment analysis. In the BP category, the small molecule catabolic, alpha–amino acid biosynthetic, and small molecule biosynthetic processes exhibited considerable enrichment (Figure 5A,B). In the CC category, the mitochondrial matrix, myosin complex, and cytosol exhibited remarkable enrichment (Figure 5A,C). In the MF category, heterocyclic and organic cyclic compound binding exhibited substantial enrichment (Figure 5A,D).

### 2.3 Kyoto Encyclopedia of Genes and Genomes pathway annotation and enrichment of differential proteins

**FIGURE 6:**
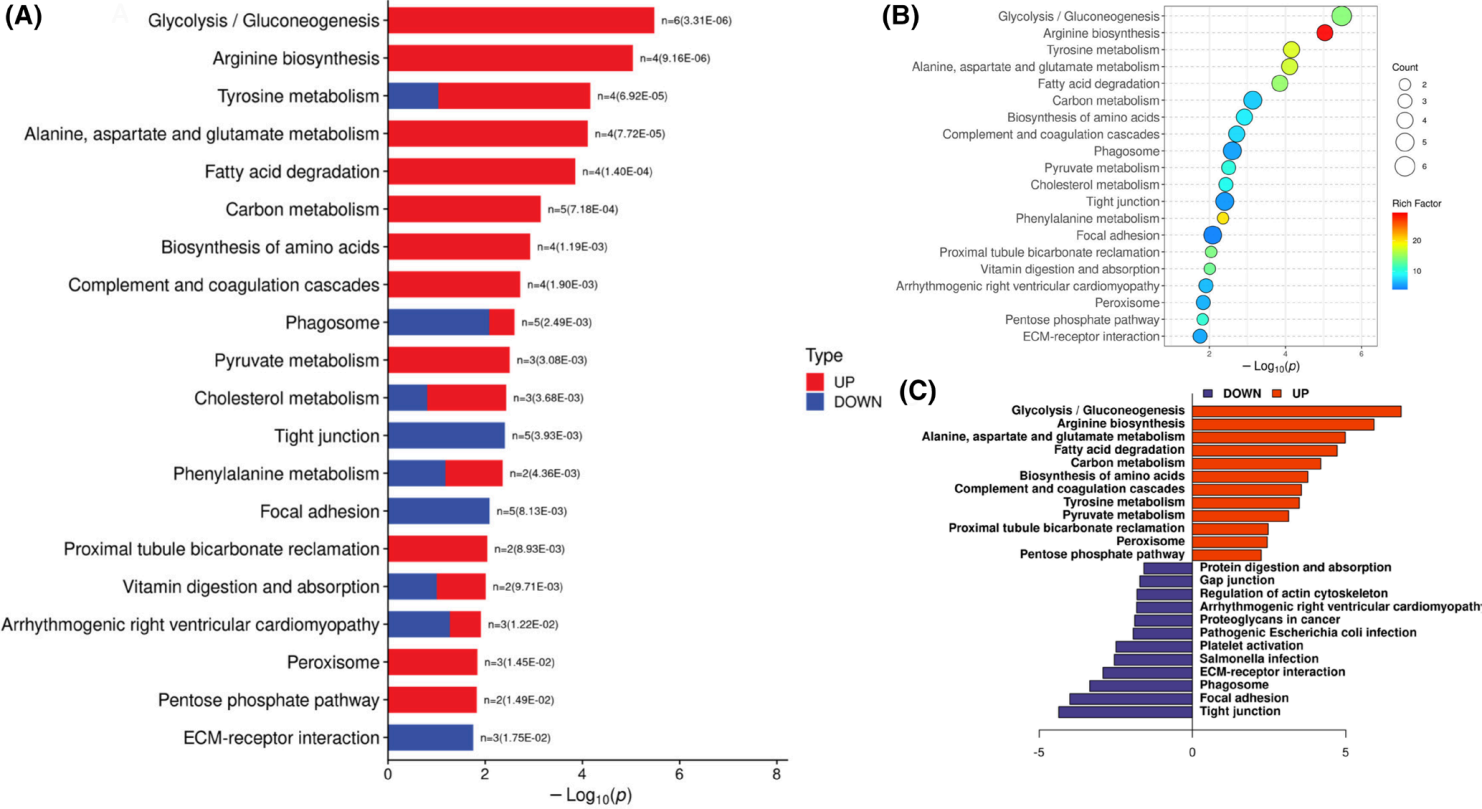
KEGG pathway annotation and enrichment of differential proteins. (A) KEGG pathway enrichment bar graph of differential proteins (*p* value, down). (B) Enrichment bubble diagram of KEGG pathway of differential protein (top 20). (C) Butterfly diagram of the upregulated and downregulated proteins with regard to KEGG pathway enrichment.

The Kyoto Encyclopedia of Genes and Genomes (KEGG) function enrichment analysis was performed on the differential proteins of the comparison group according to the algorithm of Fisher's exact test. The results were expressed by *p* values (<0.05), indicating substantial functional enrichment, and a lower *p* value indicates a more considerable enrichment of the functional category. Figure 6A shows the KEGG pathway enrichment analysis for the top 20 significantly enriched protein sets, and the results indicate that the top six signal pathways are glycolysis, arginine biosynthesis, tyrosine metabolism, alanine, aspartate, and glutamate metabolism, fatty acid degradation, and carbon metabolism (Figure 6A–C). The enrichment bubble chart shows that glycolysis and arginine biosynthesis are the top enriched pathways (Figure 6A,B). The butterfly diagram of the enrichment of the KEGG pathway of upregulated and downregulated proteins shows that the upregulated pathways are glycolysis and arginine biosynthesis and the downregulated pathways are tight junction and focal adhesion (Figure 6A–C).

### 2.4 Arginine biosynthesis signal pathway in the central position of the protein functional interaction network

**FIGURE 7:**
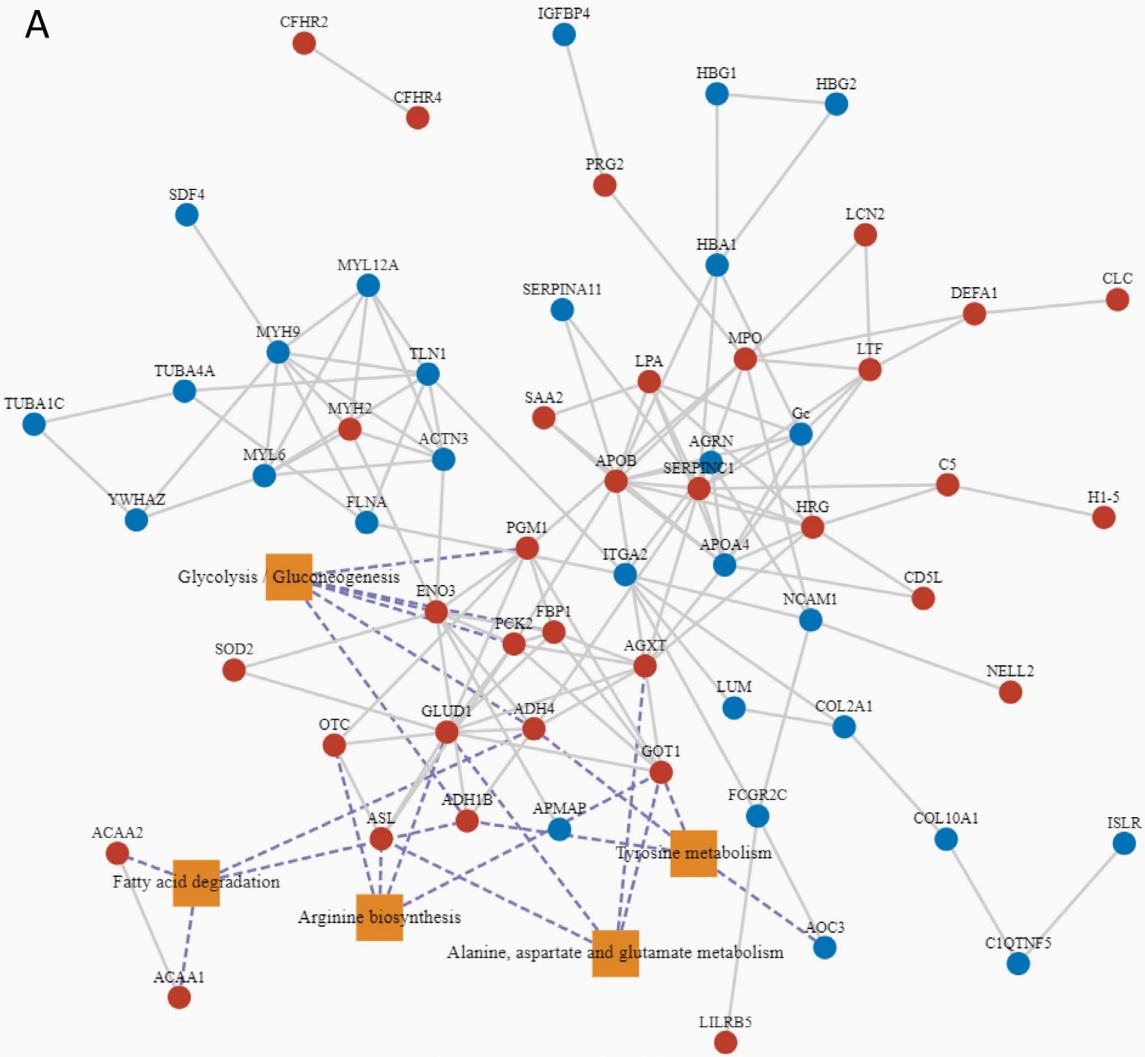
(A) Interaction between the top substantial enrichment pathway (*p* < 0.05) and considerably different proteins.

To determine regulatory proteins that play a key role in differential proteins, we combined protein–protein interaction data from the STRING database and the relationship between pathways and proteins and analyzed the differential proteins using the protein functional interaction network (PFIN). Protein interaction analysis revealed that the four proteins of the Arginine biosynthesis signal pathway (OTC, ASL, GOT1, and GLUD1) were in the center of the PFIN (Figure 7A). The heat map of gene expression exhibited a considerable increase in the expression of four genes. Our results indicate that the arginine biosynthesis signal pathway may be the therapeutic target of MeCP2 syndrome.

## 3 CONCLUSION AND RESULTS

Our discovery of a new spontaneous MeCP2 nonsense mutation enriches the understanding of Rett syndrome and provides a reference for its early diagnosis and treatment.

## 4 DISCUSSION

After birth, the patient was found to have a left‐to‐right shunt of the cardiac foramen ovale and mild regurgitation of the bicuspid and tricuspid valves. Many studies have revealed cardiac defects in mice with MeCP2 gene knockout.^10^ Therefore, we suggest that in patients with MeCP2 gene variants, early intervention can be considered once the mutation of cardiac malformation is found, rather than waiting to be observed. As the patient is still young, phenotypes such as mental retardation and autism may not be evident at this time. Studies have shown that exercise training can alleviate anxiety and prolong the lifespan of MeCP2 mutant mice.^11^ In the future, we will conduct clinical training to evaluate the therapeutic effect of exercise on MeCP2 mutant patients. Nowadays, research on MeCP2‐related disorders focuses on neurological diseases, with little attention given to other organs. MeCP2 is expressed in most tissues, with high expression in the brain, lung, and spleen and relatively low expression in the liver, heart, kidney, and small intestine. This patient has horizontal fissures, mild pulmonary effusion in the lungs, and a high level of PCO_2_. These phenotypes have not been noted before, and we will follow up on their presence in other organs.

In the MeCP2 p.Lys254* variant, the protein is truncated at amino acid 250, resulting only in one TRD domain being present in the truncated body. The C‐terminus and other TRD domains are not translated. MeCP2 can regulate the expression of many downstream genes; however, the mechanism by which MeCP2 causes the phenotype is not clear. This variant is required to study its pathogenic mechanism.

Several studies have indicated that MeCP2 mutation influences multiple metabolisms, such as fatty acid degradation, alanine aspartate, glutamate metabolism, tyrosine metabolism, and glycolysis/gluconeogenesis. Herein, in addition to the established findings, our analysis revealed considerable changes in the arginine biosynthesis pathway (OTC, ASL, GOT1, and GLUD1). PFIN analysis indicated this pathway is in the center. Moreover, we found that the expressions of ACTN3, cacna2d1, and TGA2, all associated with arrhythmogenic right ventricular cardiomyopathy, were considerably altered, which explains cardiac defects in some patients. Our results indicated that these proteins may be the therapeutic target of MeCP2 syndrome.

## AUTHOR CONTRIBUTIONS

*Conceptualization*: Xiangwen Peng and Xiaoqin Gong, Anji Chen. *Methodology*: Tuanmei Wang and Jianxin Gao. *Validation*: Baomei Yu, Jun He. *Formal analysis*: HXQ. *Investigation*: Yunsu Zhu *Writing*—*original draft preparation*: Xiangwen Peng, Min Wu, and Tuanmei Wang. *Writing*—*review and editing*: Jinjin Dai. All authors have read and agreed to the published version of the manuscript.

## FUNDING INFORMATION

This research supported by Clinical Medical Technology Demonstration Base for Genetic Research of Fetal Congenital Heart Disease in Hunan Province (2021SK4036), Hunan Province Children's Safe Medication Clinical Medical Technology Demonstration Base(2023SK4083), Project of Changsha Science and Technology Bureau, (KH2201045), Natural Science Foundation of Hunan Province (2023JJ30063); Changsha Science and Technology Bureau natural science surface project (kq2202030) and National Natural Science Foundation of China (32070817).

## CONFLICT OF INTEREST STATEMENT

The authors declare no conflict of interest.

## CONSENT

Written informed consent was obtained from the patient to publish this report in accordance with the journal's patient consent policy.

## INSTITUTIONAL REVIEW BOARD STATEMENT

This study was conducted according to the guidelines of the Declaration of Helsinki and approved by the Ethics Committee of Changsha Maternal and Child Health Hospital.

## Data Availability

The data that support the findings of this study are available on request from the corresponding author. The data are not publicly available due to privacy or ethical restrictions.
